# Bridging the scales for deformable red blood cells simulation: from resolved immersed boundary to unresolved CFD–DEM

**DOI:** 10.1007/s10237-026-02071-9

**Published:** 2026-06-02

**Authors:** Carmine Porcaro, Jonas Latt, Mahdi Saeedipour

**Affiliations:** 1 Department of Particulate Flow Modelling, Johannes Kepler University, Linz, Austria; 2 Department of Computer Science, University of Geneva, Geneva, Switzerland

**Keywords:** Blood flow simulation, Upscaling strategy, Red blood cells mechanics, Lattice Boltzmann Immersed Boundary, Unresolved CFD–DEM method

## Abstract

Several methods exist for cell-level simulation of blood, due to the undeniable interest this topic holds in the biomedical field. Nevertheless, the amount of computational resources required for these resolved methods limits their application to large-scale problems. On the other hand, the single-phase approximation of blood as a non-Newtonian liquid, while attaining fast results, neglects its multiphase nature, hindering its application to all cases where the presence of cells cannot be overlooked. This work aims to bridge the gap between these two approaches by proposing an efficient upscaling strategy. An immersed boundary approach based on the lattice Boltzmann method (LBM), coupled with a nodal projective finite element solver (npFEM) for the dynamics of the red blood cell (RBC) membrane is adopted to construct a dataset for the properties of RBCs embedded in a flow. Different quantities such as RBC deformation and experienced drag and lift forces are then collected and utilized to derive closure models for an unresolved CFD–DEM solver, which allows the simulation of hundreds of thousands of RBCs with substantially lower computational costs. The strategy is tested against previous experimental studies and then used for real-scale applications such as the design of microfluidics devices for plasma separation and the investigation of channels with different degrees of stenosis mimicking cases of vein occlusion. The presented approach potentially allows cell-level blood flow simulations with up to O$(10^6)$ RBCs and returns accurate results when tested in scenarios including hundreds of thousands RBCs, as proven by our prior work. The original contribution of this upscaling approach is to bridge the scale separation gap in cell-level blood flow simulations. It holds considerable potential for the simulation of practical bio-physical problems, such as thrombus formation and the investigation of RBCs as biocompatible drug carriers.

## Introduction

Numerical simulation of blood flow is a promising tool to explore the complex physical properties of this biological fluid. Due to its fundamental role in the human body, a variety of simulation methods has been proposed in the literature. For applications whose domains belong to the range of millimeters, it is common to assume that blood is a single-phase, non-Newtonian fluid to avoid exponentially increasing computational costs. In this case, the usual choices are the finite volume method (FVM, Yan et al. (2020)) and finite element method (FEM, Elhanafy et al. (2019); Giannokostas et al. (2021). Nevertheless, blood is composed of 45% biological cells, with approximately 44% represented by red blood cells (RBCs), and the remaining part split between white blood cells (WBCs) and platelets. Therefore, several biological phenomena are linked to this multiphase nature, such as platelet margination (Aarts et al. 1988) and the Fåhraeus–Lindqvist effect (Fåhraeus and Lindqvist 1931). Furthermore, accounting for the presence of RBCs is unavoidable when simulating many specific biomedical applications, like microfluidic separation of blood components (Hur et al. 2011; Catarino et al. 2019), diagnosis of malaria infection due to RBC hardening (Ademiloye et al. 2017), intracellular drug delivery through RBCs (Bernardelli et al. 2025) or microvascular network dynamics (Ye et al. 2019; Li et al. 2020, 2023). This explains the need for cell-level simulation, at the price of rising the computational cost due to the costly numerical resolution of membrane dynamics together with cell–cell interactions. Examples in the literature include: meshless methods such as boundary element method (BEM) (Lu and Peng 2019; Rydquist and Esmaily 2022, 2023), smoothed particle hydrodynamics (SPH) (Soleimani et al. 2019) and dissipative particle dynamics (DPD) (Pan et al. 2010; Fedosov et al. 2010) or Immersed Boundary Methods (IBM) (Krüger et al. 2014; Cimrák et al. 2014; Závodszky et al. 2017; Kotsalos et al. 2019; Lehmann et al. 2020). The authors have previously introduced an IBM approach using a reduced-order model for the RBCs, applying it to hemolysis prediction in microfluidic devices with a physiological 40% hematocrit (Nair et al. 2020; Nair 2021; Nair et al. 2022; Porcaro and Saeedipour 2023). Nevertheless, due to the long computational times required by all the aforementioned methods, cell-level numerical simulation is usually either limited to small domains and low hematocrits, or demands very large computational resources.

Recently, different works have been trying to bridge the gap between the continuum and the fully resolved particle scale. One option is represented by Lagrangian-tracking algorithms promptly adapted to resolve particle deformability at the cellular level (Wedel et al. 2024; Mansour et al. 2025). These methods are based on a one-way coupling approach in which the fluid flow is resolved and then used to compute RBC properties (position, shape, cell damage). This allows for a considerable upscaling of the simulation domain, without compromising the accuracy of representation of cell-level RBC properties. Furthermore, for the specific application of hemolysis prediction, Dirkes et al. (2024) proposed an Eulerian formulation of this concept, eliminating the need for Lagrangian particle tracking. The drawback of these approaches is that they neglect the interactions among RBCs and their influence on the fluid flow, which might be crucial in conditions of high hematocrits. On the other hand, the authors have introduced a new simulation technique based on an unresolved Computational Fluid Dynamics and Discrete Element Method coupling (unresolved CFD–DEM, Porcaro and Saeedipour (2024)), which allowed the simulation of half a million RBCs using only 8 processors while still accounting for a full fluid-cell coupling and cell-cell interactions. This comes at the cost of unresolved membrane dynamics, and a need for closure models for different variables such as drag and lift forces acting on RBCs, RBC deformation, and orientation. Despite producing results in line with previous literature data about macroscopic blood characteristics in channel flow (relative apparent viscosity $\mu _\mathrm{{{rel}}}$ and discharge hematocrit $Ht_d$), the model suffered from an inherent uncertainty regarding specific features of immersed RBCs, including their deformation and orientation, because the dataset used for developing these closure expressions was based on an RBC reduced-order model.

**Fig. 1:**
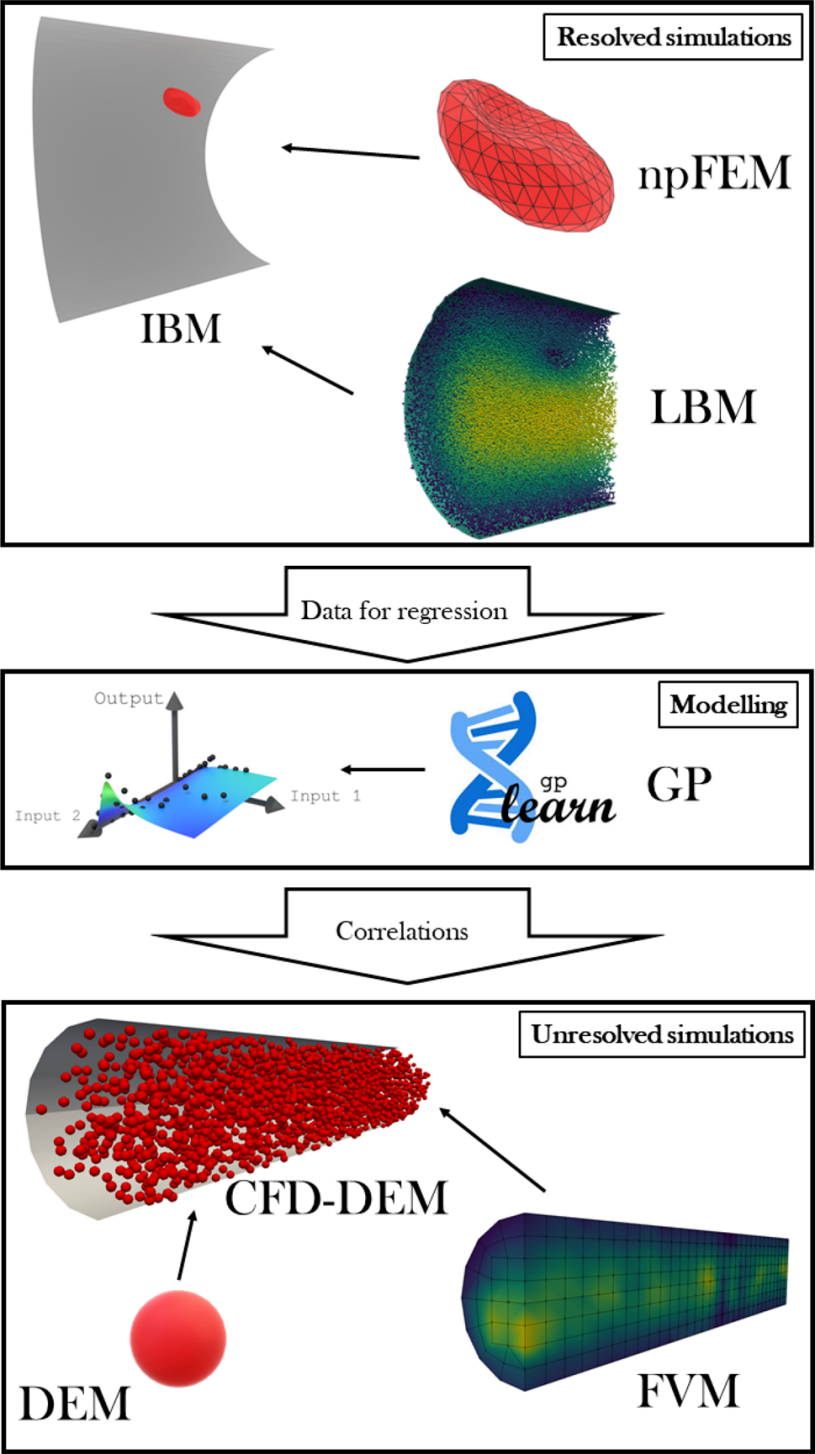
Summary of the upscaling approach implemented in this work. A resolved simulation campaign is carried out to gather data about the RBC dynamics in flow. This dataset is then used to build models through a regression algorithm. The models are then used in the unresolved simulations of RBC-laden flow

To overcome these limitations, a follow-up to the previous work is presented in this paper. A coupled lattice Boltzmann method (LBM) and nodal projective finite element method (npFEM) solver (Kotsalos et al. 2019) is used to simulate the dynamics of individual RBCs in a flow. This method resolves the membrane dynamics of RBCs physically accurate fashion, addressing both the need to refine the data accuracy and to include additional details into the CFD–DEM model. This leads to a more complete description of the RBC behavior in a flow, improving the results on large-scale simulations. Once the required dataset was built, it could be used to identify the correlations between fluid properties at the macroscale (i.e., fluid velocity, relative velocity between fluid and particles, shear gradient) and the needed microscale variables. This purpose was achieved following a genetic programming approach. The models were then applied to the unresolved CFD–DEM simulations of blood flow. The upscaling workflow is summarized in Fig. 1. The validity of the proposed strategy is demonstrated through comparison with experimental data, together with its utility in studying different types of biomedical problems.

It should be noted that this work represents a first step toward cell-level simulation of blood flow on a scale comparable to experimental conditions, but still presents different limitations related to the assumptions used in the modeling phase. First, the closure models are based on resolved simulations of a single RBC, which affects the accuracy of the method for dense suspensions, e.g., hematocrits above 15%, as proven in authors’ previous work (Porcaro and Saeedipour 2024). Furthermore, the range of analyzed Reynolds numbers is kept below one, thus hindering the reliability of the models for flows with higher inertial effects. Finally, a model for the orientation of the red blood cells is not included in this work, since in many of the resolved simulations a tumbling behavior was observed, showing no preferred inclination of the RBC compared to the flow. More details on each of these limitations can be found in the dedicated paragraphs in the text. Nevertheless, in the regime of low Reynolds number flows with sufficiently low hematocrits, the proposed upscaling method shows satisfying agreement with experimental works. It underlines that with a more extended modeling phase, this numerical tool could be employed as a complement to in vitro studies for bio-physical applications on a realistic scale.

## Numerical methods

This section will briefly explain the numerical tools used for this work. The LBM-npFEM code used for single RBC simulations has been extensively validated in previous works (Kotsalos et al. 2019, 2022), as well as the CFD–DEM method (Porcaro and Saeedipour 2024). Hence, for detailed explanations of the two approaches, the reader is referred to the aforementioned literature. Although the two methods exhibit fundamental differences, they both aim at resolving the equations of motion for a suspension of deformable bodies (RBCs) in a carrier fluid (plasma). The dynamics of the blood plasma is described by the incompressible Navier–Stokes equations (NSE).

1 $$\begin{aligned} &  \nabla \cdot {\textbf {u}} = 0 \end{aligned}$$

2 $$\begin{aligned} &  \rho \Bigl (\frac{\partial ({\textbf {u}})}{\partial t} + \nabla \cdot ({\textbf {u}} {\textbf {u}})\Bigr ) = - \nabla p + \mu \nabla ^2 {\textbf {u}} \end{aligned}$$

which, in the LBM-npFEM approach, are solved with help of the lattice Boltzmann method (LBM) for weakly compressible fluids (Krüger et al. 2017), while the CFD–DEM approach solves the NSE directly using a finite volume method (FVM). In both methods, a slight modification of Eq. (1) and Eq. (2) is required to account for the presence of solid particles in the domain, as explained in the following paragraphs.

### Resolved simulations

An IBM combining an LBM solver for the fluid part and an npFEM solver for the deformable body was employed to build a reliable dataset for the modeling aspects of this work. Paragraphs 2.1.1 and 2.1.2 explain the mathematical formulation and implementation of the two solvers, while paragraph 2.1.3 briefly introduces the IBM.

#### Lattice Boltzmann method

The LBM was originally developed as a follow-up to lattice gas models. It also has a strong link with the kinetic theory of gases, from which it inherits as a fundamental quantity the velocity distribution function $f_i({\textbf {x}}, t)$ (also known as a particle population). The main difference with lattice gas models lies in the fact that, instead of tracking the particles directly, LBM tracks their statistical distribution in phase space. $f_i({\textbf {x}}, t)$ represents the probability density function of particles with velocity $\{{\textbf {c}}_i\}^{q-1}_{i=0}$ at position ${\textbf {x}}$ and time *t*, with *q* being the number of populations of a single lattice node related to the discretization of the velocity space. A spatial discretization is also applied, with a regular distribution of points in space, located at discrete positions x, and with a distance $\Delta x$ between adjacent points. An LBM iteration cycle is based on two steps: a local collision step and a non-local streaming step, during which the populations are copied to neighboring nodes. A collision-streaming cycle advances the state of the system from time *t* to $t + \Delta t$, where $\Delta t$ is the simulation time-step. The two steps are represented, respectively, by the following equations:

3 $$\begin{aligned} &  f_i({\textbf {x}}, t) \leftarrow f_i({\textbf {x}}, t) - \frac{f_i({\textbf {x}}, t) - f_i^{eq}({\textbf {x}}, t)}{\tau } \Delta t \end{aligned}$$

4 $$\begin{aligned} &  f_i({\textbf {x}} + {\textbf {c}}_i \Delta t, t + \Delta t) = f_i({\textbf {x}}, t) \end{aligned}$$

In Eq. (3), the so-called Bhatnagar-Gross-Krook (BGK) collision operator was used (Bhatnagar et al. 1954). This operator relaxes the population toward equilibrium with a certain relaxation time $\tau$. The equilibrium distribution function $f_i^{eq}({\textbf {x}}, t)$ depends on the local fluid density and velocity $\rho$ and ${\textbf {u}}$, which could be calculated as:

5 $$\begin{aligned} &  \sum _i f_i({\textbf {x}}, t) = \rho \end{aligned}$$

6 $$\begin{aligned} &  \sum _i {\textbf {c}}_i f_i({\textbf {x}}, t) = \rho {\textbf {u}} \end{aligned}$$

It also depends on the isothermal speed of sound $c_s$, which is set to:

7 $$\begin{aligned} c_s = \frac{1}{3} \frac{(\Delta x)^2}{(\Delta t)^2} \end{aligned}$$

in the D3Q19 velocity set used in this work. This entails that the velocity space is discretized in three dimensions and with $q = 19$ velocities. Each velocity set is composed of a collection of velocities ${\textbf {c}}_i$ and weighting coefficients $w_i$ (for further details on velocity sets, see Krüger et al. 2017, paragraph 3.4.7). The equilibrium distribution function can be finally characterized as:

8 $$\begin{aligned} f_i^{eq}({\textbf {x}}, t) = w_i \rho \left( 1 + \frac{{\textbf {u}} \cdot {\textbf {c}}_i}{c_s^2} + \frac{({\textbf {u}} \cdot {\textbf {c}}_i)^2}{2c_s^4} - \frac{{\textbf {u}} \cdot {\textbf {u}}}{2c_s^2} \right) \end{aligned}$$

Through the so-called Chapman-Enskog analysis it is possible to show that the fluid-dynamic behavior predicted by LBM equations is equivalent to the one predicted by NSE when relaxation time and kinematic viscosity of the fluid are linked in the following way:

9 $$\begin{aligned} \nu = c_s^2 \left( \tau - \frac{\Delta t}{2} \right) \end{aligned}$$

Although not directly necessary for solving the lattice Boltzmann equations, it is also possible to recover the hydrodynamic stress tensor through the following equation:

10 $$\begin{aligned} \boldsymbol{\sigma } = -p \boldsymbol{I} + \left( \frac{1}{2\tau } - 1 \right) \sum _i \boldsymbol{c_i c_i} (f_i - f_{eq}) \end{aligned}$$

The lattice Boltzmann equations are solved in this work using the open-source code Palabos (Latt et al. 2021).

#### Nodal projective finite element method

**Fig. 2:**
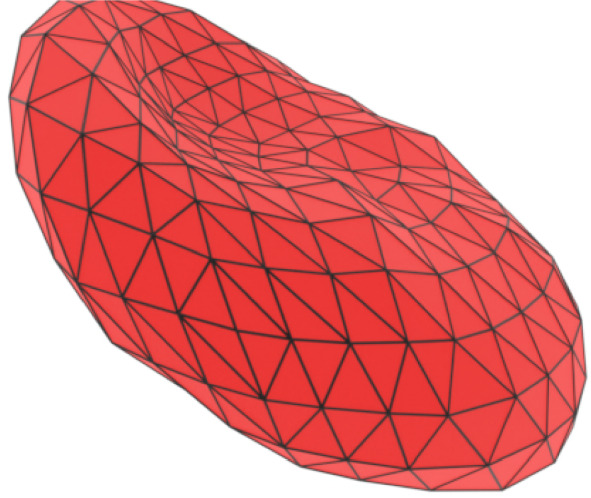
Representation of the RBC membrane discretized in $n_{vert}$ vertices in the npFEM solver

The npFEM has been previously used to simulate hyperelastic materials (Bouaziz et al. 2014; Liu et al. 2017). Its adaptation to the description of RBC dynamics is thoroughly explained in Kotsalos et al. (2019). Thus, this paragraph will only serve as a short introduction to the approach. The RBC is represented by a triangulated membrane, with its total mass (including the internal fluid), lumped into the vertices. A position and a velocity vector (respectively $\boldsymbol{x_{vert}}$ and $\boldsymbol{u_{vert}}$) are assigned to each of the $n_{vert}$ vertices (see Fig. 2). It is possible to distinguish two types of forces acting on them, namely external forces $F_{ext}$ coming from the fluid-solid interaction, and internal forces $F_{int}$, which are position-dependent and linked to the sum of potential energies acting on that vertex:

11 $$\begin{aligned} \boldsymbol{F_{int}}(\boldsymbol{x_{vert}}) = - \sum _i \nabla E_{pot,i} (\boldsymbol{x_{vert}}) \end{aligned}$$

The equations to advance vertices positions and velocities to a subsequent time-step are based on an implicit Euler time integration scheme:

12 $$\begin{aligned} &  \boldsymbol{u^{n+1}_{vert}} = \frac{\boldsymbol{x^{n+1}_{vert}} - \boldsymbol{x^{n}_{vert}}}{\Delta t} \end{aligned}$$

13 $$\begin{aligned} &  \boldsymbol{F_{int}(x^{n+1}_{vert}) + F_{ext}(x^n_{vert}) - C u^{n+1}_{vert}} = M \frac{\boldsymbol{u^{n+1}_{vert}} - \boldsymbol{u^{n}_{vert}}}{\Delta t} \nonumber \\ \end{aligned}$$

where ${\textbf {M}}$ is the mass matrix and ${\textbf {C}}$ is a damping matrix used for Rayleigh damping, which mimicks the viscoelastic behavior of the RBC membrane. The two equations could be combined to give a minimization problem, whose solution is $\boldsymbol{x^{n+1}_{vert}}$. For specific details on the used potential energy, on the material constitutive equations, on Rayleigh damping, and in general on the npFEM algorithm, the reader is referred to Kotsalos et al. (2019).

#### Immersed boundary method

The Immersed Boundary Method was developed by Peskin (1972), to study blood flow around heart valves. Since then, it has been applied to several topics, since its formulation can be generalized to a wide range of numerical simulation methods (Mittal and Iaccarino 2005). Compared to approaches using body conformal meshes, it is easier to implement and has a lower computational cost, although slightly compromising on accuracy. IBM is used in this work to couple the deformable body dynamics solved by the npFEM method to the fluid dynamics solved through LBM. This entails a modification of fluid velocity (found through Eq. (6)) based on a forcing term $\boldsymbol{F_{imm}}$ applied as a body force to the fluid. This is equivalent to imposing a no-slip condition at the boundary between solid and fluid. Algorithm 1 explains the IBM steps. The computation of $\boldsymbol{F_{imm}}$ is based on an iterative procedure described in Kotsalos et al. (2019), where the velocity of the fluid in the Eulerian reference frame is corrected based on an interpolation of the velocity of the Lagrangian points describing the solid membrane. This term could also be used as $\boldsymbol{F_{ext}}$ in Eq. (13), but for higher accuracy this second force is directly computed based on the stress applied from the fluid (Eq. (10)).
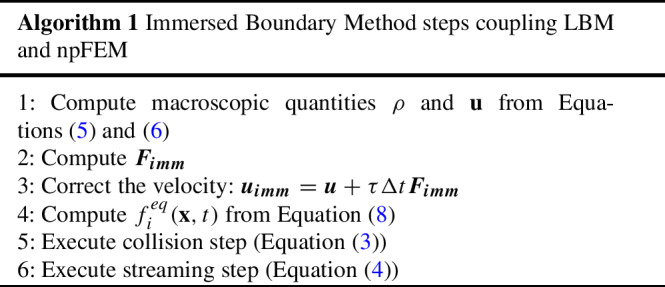


### Unresolved simulations

This section will introduce the CFD–DEM method, which combines an FV solver for the fluid part and a DEM approach for the solid particles. A more detailed description could be found in Goniva et al. (2012). In this approach, RBCs are subgrid-scale entities represented by a sphere of equivalent volume. For this reason, different microscale properties (e.g., forces acting on the particles, deformation, orientation) have to be coarse-grained to the grid scale and rely on adequate closure laws. The CFD–DEM solver is used for the efficient unresolved simulation of hundreds of thousands of RBCs, based on models that are derived from the LBM-npFEM resolved simulations. Specific care is needed for the time-discretization of the equations since the CFD and DEM parts both require some criteria to be fulfilled in order to ensure the stability and accuracy of the simulations. The time-step for FV simulation $\Delta t_{CFD}$ is strictly connected to the size of the FV mesh grid $\Delta x_{CFD}$, through a Courant number $Co = \frac{u_{max} \Delta t_{CFD}}{\Delta x_{CFD}}$ which must be smaller than unity (although it is normally kept in the $0.1-0.2$ range). $\Delta t_{DEM}$ is selected instead as $\Delta t_{DEM} < 0.1~min(t_{Ray}, t_{Her})$, where $t_{Ray}$ is the Rayleigh time (Li et al. 2005) and $t_{Her}$ is the Hertz time (Landau and Lifshitz 1986). The two time steps are linked by a coupling interval $n_{coupling} > 0$, which represents the number of DEM steps in between two CFD steps: $\Delta t_{CFD} = n_{coupling}\Delta t_{DEM}$. Therefore, particle positions and velocities are updated for a certain number $n_{coupling}$ of steps before the new update of the flow field conditions.

#### Finite volume method

An FVM code (the open-source software OpenFOAM) is used to solve the incompressible version of NSE (Eqs. (1) and (2)) with slight modifications to account for a certain volume fraction of solid $\phi$ in the computational mesh cell:

14 $$\begin{aligned} &  \frac{\partial \phi }{\partial t} + \nabla \cdot \phi {\textbf {u}} = 0 \end{aligned}$$

15 $$\begin{aligned} &  \rho \Bigl (\frac{\partial (\phi {\textbf {u}})}{\partial t} + \nabla \cdot (\phi {\textbf {u}} {\textbf {u}})\Bigr ) = - \phi \nabla p + \phi \mu \nabla ^2 {\textbf {u}}-{\textbf {R}}_{\textbf {p,f}} \end{aligned}$$

The term ${\textbf {R}}_{\textbf {p,f}}$ represents the momentum exchange between fluid and particles. Details on its computation can be found in paragraph 2.2.3, dedicated to the coupling between CFD and DEM. A PISO algorithm is employed for the pressure velocity coupling (Issa 1986).

#### Discrete element method

DEM is used to update particle positions and velocities, solving two equations for conservation of linear and angular momentum:

16 $$\begin{aligned} &  m_p \frac{\partial {\textbf {u}}_{\textbf {p}}}{\partial t} = {\textbf {F}}_{\textbf {p,TOT}} = m_p {\textbf {g}} + {\textbf {F}}^{\textbf {f}}_{\textbf {p}}\nonumber \\  &  \quad + \sum _{N_p} {\textbf {F}}^{\textbf {p}}_{\textbf {p}} + \sum _{N_w} {\textbf {F}}^{\textbf {w}}_{\textbf {p}} + {\textbf {F}}^{\textbf {ext}}_{\textbf {p}} \end{aligned}$$

17 $$\begin{aligned} &  I_p \frac{\partial \boldsymbol{\omega }_{\textbf {p}}}{\partial t} = {\textbf {r}} \times {\textbf {F}}_{\textbf {p,TOT}} = {\textbf {r}}\nonumber \\  &  \quad \times \biggl ( {\textbf {F}}^{\textbf {f}}_{\textbf {p}} + \sum _{N_p} {\textbf {F}}^{\textbf {p}}_{\textbf {p}} + \sum _{N_w} {\textbf {F}}^{\textbf {w}}_{\textbf {p}} + {\textbf {F}}^{\textbf {ext}}_{\textbf {p}} \biggr ) \end{aligned}$$

$m_p$ and $I_p$ are respectively the particle mass and moment of inertia. **g** is the gravitational acceleration, while ${\textbf {F}}^{\textbf {f}}_{\textbf {p}}$, ${\textbf {F}}^{\textbf {p}}_{\textbf {p}}$, ${\textbf {F}}^{\textbf {w}}_{\textbf {p}}$, and ${\textbf {F}}^{\textbf {ext}}_{\textbf {p}}$ are particle-fluid, particle-particle, particle-wall, and particle-external source interaction forces. Particle-particle interactions are handled through a soft-sphere approach, allowing a small overlap between them in order to compute the resulting interaction force (Goniva et al. 2012). In the unresolved approach, the fluid-particle force interaction is split into four terms:

18 $$\begin{aligned} {\textbf {F}}^{\textbf {f}}_{\textbf {p}} = {\textbf {F}}^{\textbf {f}}_{\textbf {p,v}} + {\textbf {F}}^{\textbf {f}}_{\textbf {p,p}} + {\textbf {F}}^{\textbf {f}}_{\textbf {p,drag}} + {\textbf {F}}^{\textbf {f}}_{\textbf {p,lift}} \end{aligned}$$

with ${\textbf {F}}^{\textbf {f}}_{\textbf {p,v}}$, ${\textbf {F}}^{\textbf {f}}_{\textbf {p,p}}$, ${\textbf {F}}^{\textbf {f}}_{\textbf {p,drag}}$, and ${\textbf {F}}^{\textbf {f}}_{\textbf {p,lift}}$ being respectively the viscous, pressure, drag and lift force acting on the particle. For the mathematical treatment of the first two forces, the reader is referred to Goniva et al. (2012). The authors recently developed specific drag and lift force models for the simulation of RBCs in low Reynolds number conditions (Porcaro and Saeedipour 2024). As mentioned in the Introduction, the main purpose of the current work is to improve and extend these models through the usage of more accurate data based on resolved simulations employing the LBM-npFEM code. Algorithm 2 summarizes the DEM steps between two subsequent CFD steps. The open-source solver LIGGGHTS accounts for the simulation of the solid particles.
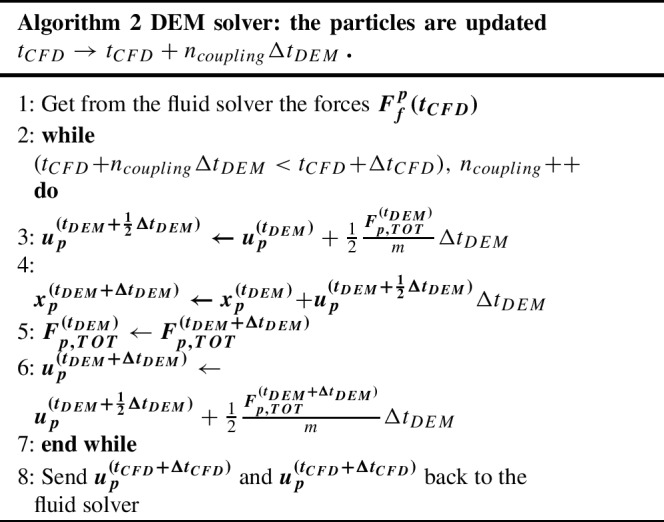


#### CFD–DEM algorithm

**Fig. 3:**
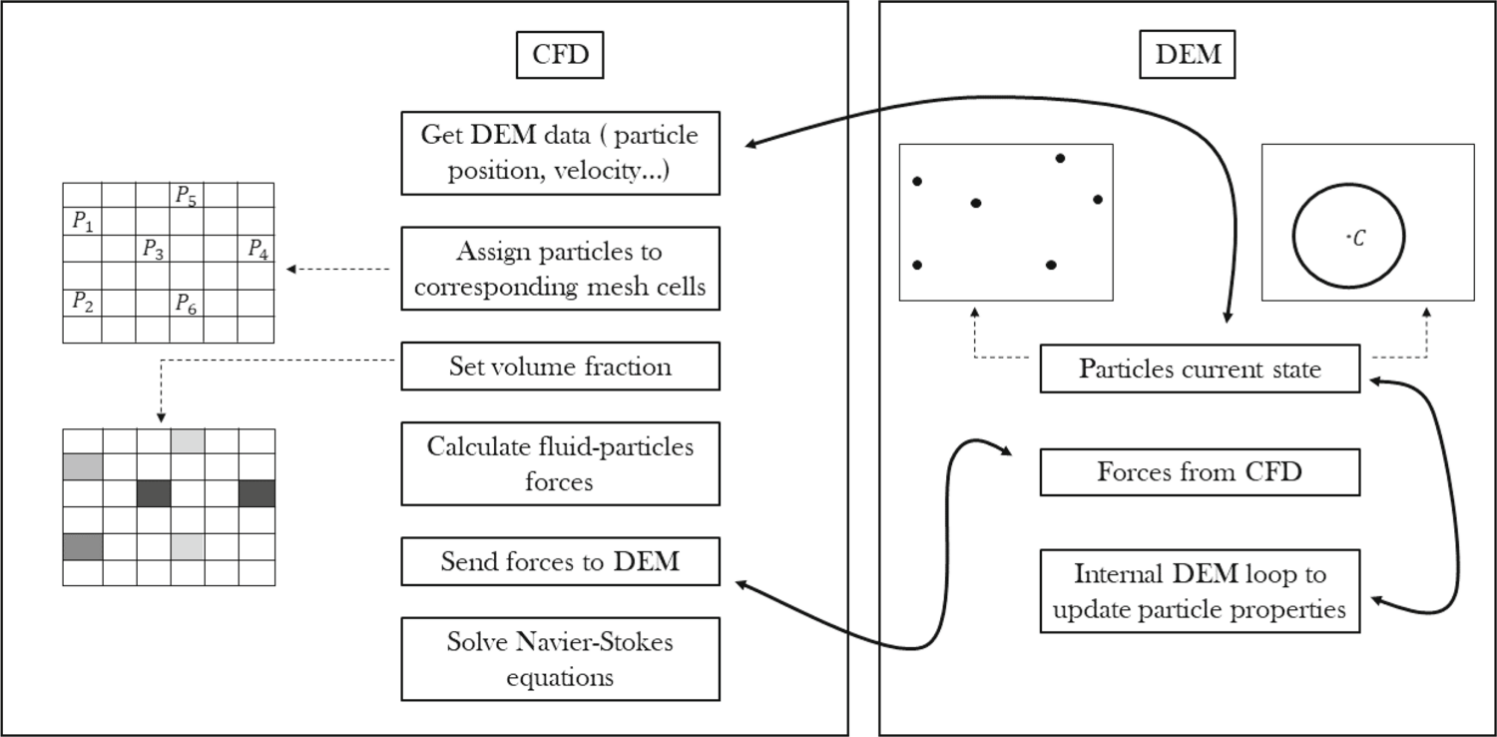
Schematic explanation of a single time-step algorithm in unresolved CFDEMcoupling. Solid arrows represent the communication between the CFD and DEM sides at a specific point in the algorithm. Dashed arrows guide to the schematic illustration of certain stages

Algorithm 3 sums up the coupling steps between the solid and fluid part in the CFD–DEM method. This is handled through the open-source software CFDEMcoupling. The main purpose of CFDEMcoupling is to coordinate the communication between the CFD and DEM parts, allowing for the necessary information to be passed to the other side. Figure 3 also gives a visual representation of these steps.
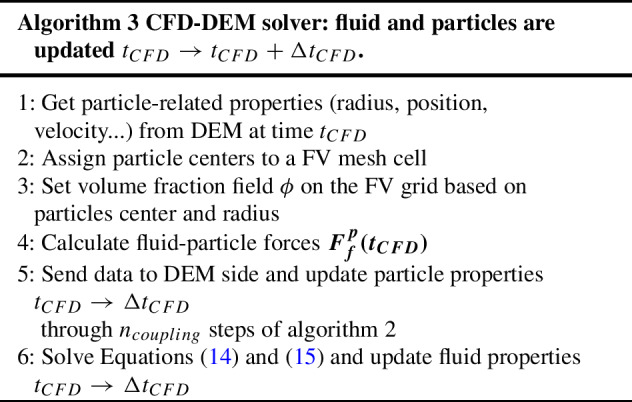


The term ${\textbf {R}}_{\textbf {p,f}}$ mentioned in paragraph 2.2.1 represents the momentum exchange between fluid and particles, and it is divided into an implicit and an explicit part for numerical reasons:

19 $$\begin{aligned} {\textbf {R}}_{\textbf {p,f}} = K_{p,f} {\textbf {u}}-K_{p,f} \langle {\textbf {u}}_{\textbf {p}} \rangle \end{aligned}$$

20 $$\begin{aligned} K_{p,f} = \frac{|\sum _i ({\textbf {F}}^{\textbf {f}}_{\textbf {p,drag}} + {\textbf {F}}^{\textbf {f}}_{\textbf {p,lift}})|}{V_{cell} \cdot |{\textbf {u}}- \langle {\textbf {u}}_{\textbf {p}} \rangle |}, \end{aligned}$$

where $V_{cell}$ is the volume of the computational grid cell. The employed coupling approach has been extensively used over the years for different applications, even outside the framework of CFDEMcoupling (Capecelatro et al. 2015). An extended overview of the state of the art of the coupling approach can be found in the recent work of (Esgandari and Schneiderbauer 2025), while fundamental concepts can be retrieved from (Zhou et al. 2010).

**Fig. 4:**
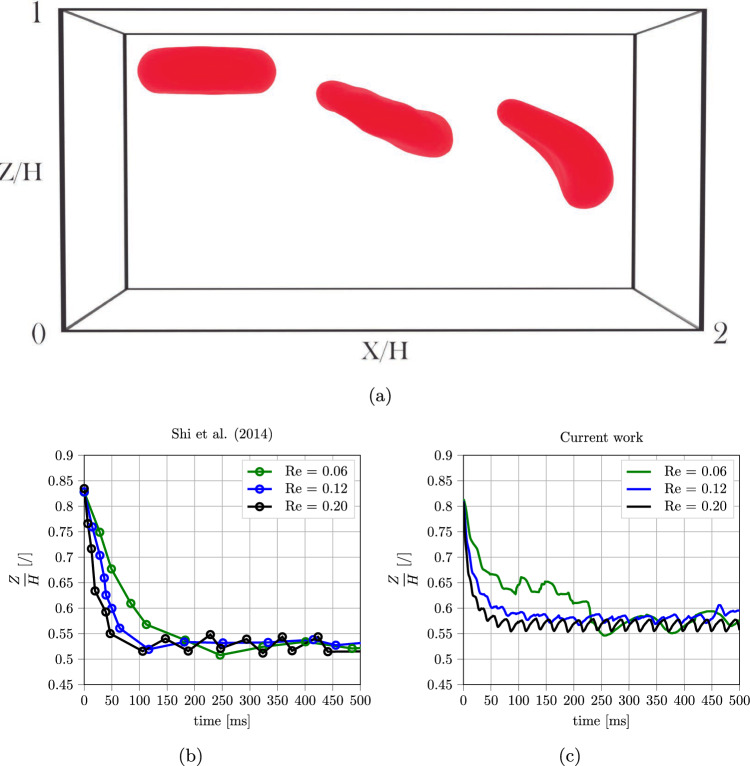
Lateral migration of an RBC in a Poiseuille flow for different *Re*. Flow is directed toward the x-axis, cross-stream migration happens over the z-axis (periodicity is imposed over the y-axis): a) snapshots of the simulations. b), c) trajectories from Shi et al. (2014) and current work

## Validation of resolved method

The LBM-npFEM approach for RBCs simulation was extensively validated in previous works (Kotsalos et al. 2019, 2022). Nevertheless, further case studies were considered to strengthen the validity of this approach for the simulation of single RBCs. The validation setups were chosen as the same as Porcaro and Saeedipour (2023), thus also allowing a comparison between the previous results obtained with a reduced-order RBC model. The RBC migration toward the center of a Poiseuille flow was first considered, followed by the deformation in a stenosed channel. Notice that it has previously been shown that the results produced by the LBM-npFEM solver are to a large extent independent of the number of vertices $n_{vert}$ used to discretize the membrane (see Kotsalos et al. 2019 for a complete mesh independence study). Therefore, this number is kept constant and equal to $n_{vert} = 258$ for the remainder of this work.

**Fig. 5:**
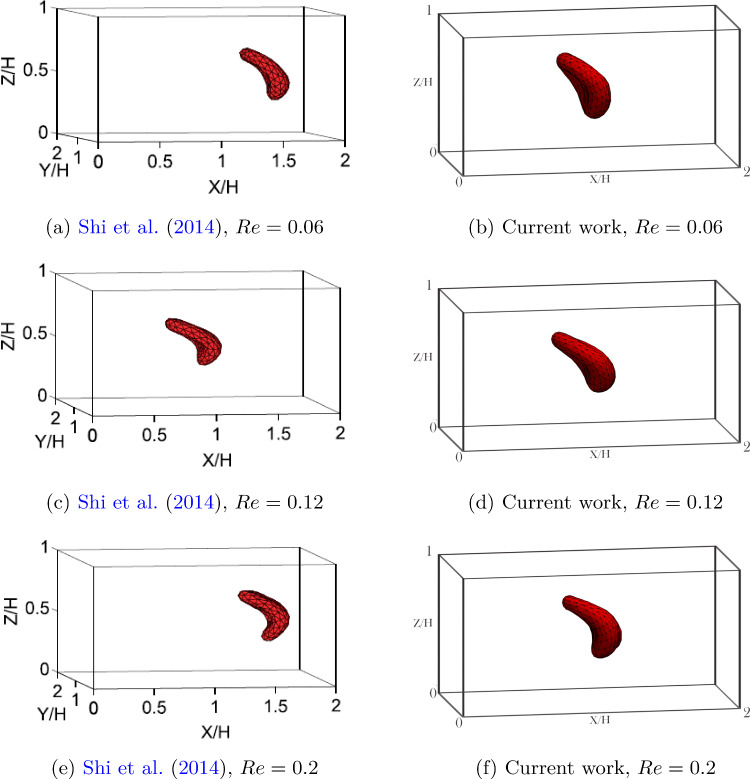
Comparison of RBC shape at the centerline after the occurrence of lateral migration for different Reynolds numbers. The left column is retrieved from Shi et al. (2014), the right column represents the current work

### RBC migration

RBCs are known to migrate toward the central axes in a confined, inertialess Poiseuille flow. This effect is caused by their deformability, which is the trigger for this kind of lateral motion even in the absence of any other activating factor (i.e., high inertia of the flow or viscoelasticity of the carrier fluid (Esposito et al. 2022; Porcaro and Villone 2023). This microscale phenomenon affects several macroscale blood properties, such as the Fåhraeus–Lindqvist effect and platelet margination. Shi et al. (2014) carried out numerical simulations in a periodic rectangular domain reproducing a Poiseuille flow condition for different Reynolds numbers $Re = \frac{\rho _f H u_{max}}{\mu }$, proving that the migration velocity is influenced by it. The simulation setup is illustrated in Fig. 4, together with simulation results obtained using different versions of the IBM: Shi et al. (2014) employed FEM for plasma and a spring network model for the RBC, while the current work used the npFEM-LBM approach described in the previous section. The latter showed a similar trend when compared to literature data, confirming the accuracy of the numerical tool. The slight difference in the equilibrium position can be attributed to the different simulation methods employed in the two works. For further details on the conditions for the validation case setup, the reader is referred to Shi et al. (2014) and Porcaro and Saeedipour (2023).

Compared to authors’ previous work with a reduced-order model for the RBC, the current approach ensures the possibility of correctly capturing also the orientation and deformation behavior of the RBC to match it to previous studies. This will be further investigated in the next test case, but can already be seen from the steady-state shape of RBCs at the end of the simulation when compared with the work of Shi et al. (2014) (see Fig. 5). In both cases, increasing the center line velocity leads to stronger deformation of the RBC after the migration has occurred. Since the RBC center of mass does not reach exactly the middle of the channel (see Fig. 4), but is slightly biased toward the direction it is coming from, the part closer to the central axis experiences higher velocity. Therefore, the final shape is similar to an asymmetrical parachute.

### RBC deformation

**Fig. 6:**
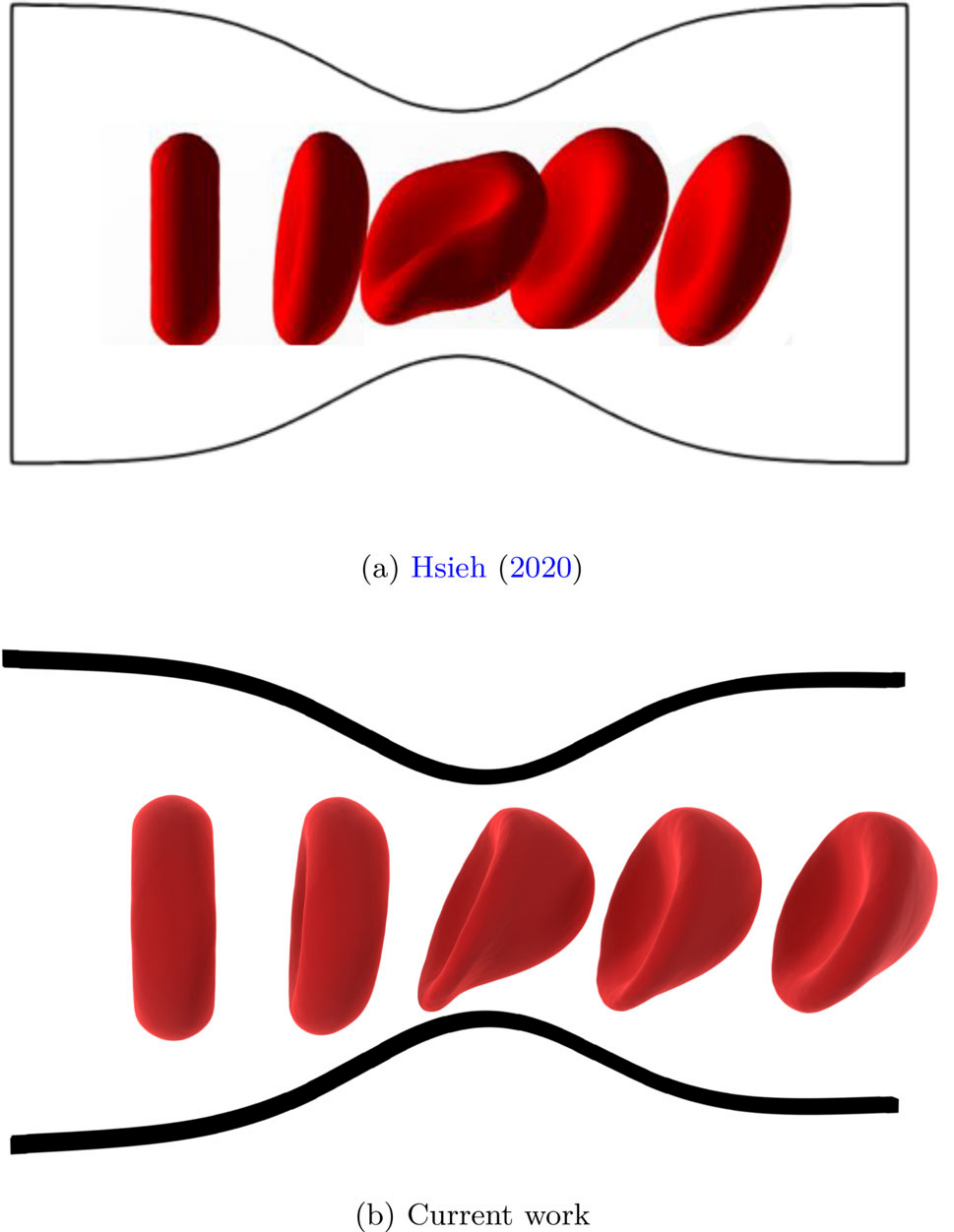
RBC passing through a stenosis, visual comparison between (Hsieh 2020) and current simulation with LBM-npFEM

**Fig. 7:**
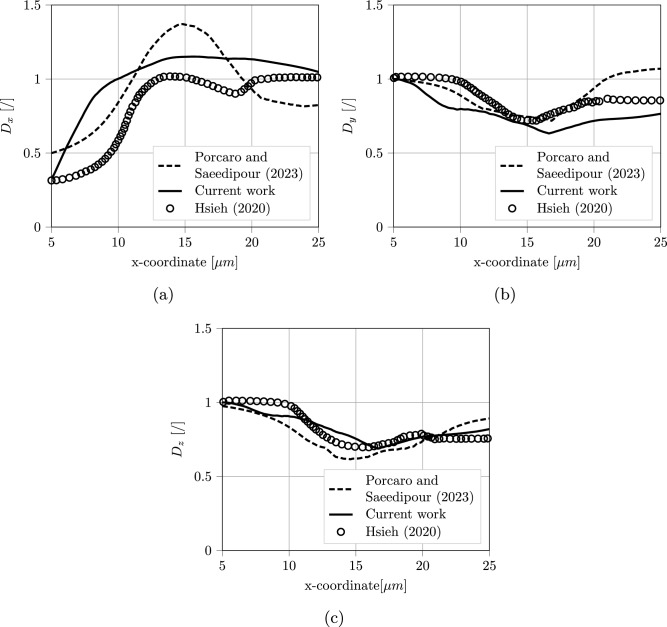
Deformation indexes in the three directions against the center of mass x-coordinate of the RBC: the flow is directed along the x-axis. Comparison among (Shi et al. 2014), Porcaro and Saeedipour (2023) and current work

RBC deformation is tested in comparison to previous work by Hsieh (2020). An RBC is placed slightly off-center in a stenosed channel, and its deformation is tracked while it passes through the constriction. This kind of geometry, characterized by a sudden contraction with subsequent expansion, recalls the real shape of arteries of patients with vascular diseases and the design of some bio-microfluidic devices (Molla and Paul 2012; Jung and Yeom 2017). Figure 6 shows a visual comparison for a minimum channel height of $8.5~\mu m$, comparing the original simulation and current work. Figure 7 illustrates the measured deformation indexes $D_x$, $D_y$ and $D_z$ in the three directions for a $5.5~\mu m$ restriction. For each direction, $D_i = \frac{L_i}{D_0}$. $L_i$ is the projected length of the RBC in the considered direction, while $D_0 = 7.82~\mu m$ is the diameter of the undeformed RBC. Results are also compared with authors’ previous work, where a more detailed description of the setup is also present. The LBM-npFEM code shows good agreement with previous literature data, ensuring its usability as a reliable source of modeling data for the unresolved simulations.

## Upscaling method

**Fig. 8:**
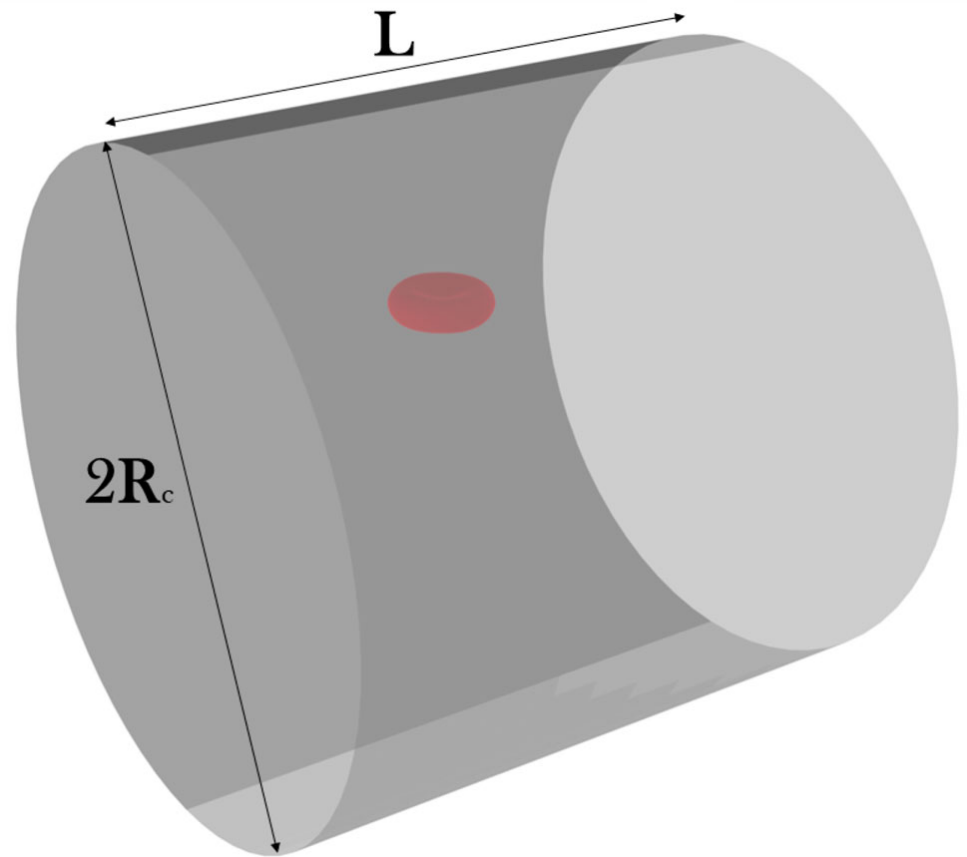
Setup for the resolved simulation of RBC using LBM-npFEM for the data collection. The RBC is located in the channel with $R_c =25~\mu m$ and $L = 50~\mu m$

As mentioned in Introduction, the purpose of this work is to derive correlations for unresolved simulation of RBCs based on highly accurate resolved data. The employed strategy is similar to Porcaro and Saeedipour (2024), but the usage of the LBM-npFEM solver allows overcoming limitations related to the reduced-order RBC model applied in the previous study. A setup with a single RBC in a cylindrical channel was used to retrieve information on different small-scale properties, such as the drag force (the force component parallel to the channel walls), the lift force oriented in the radial direction, and the membrane deformation. An example of this setup is shown in Fig. 8. To measure the drag force, the center of mass of the particle was fixed, allowing the fluid to evolve around it. For the lift force instead, following an approach used in literature for rigid particles (Yang et al. 2005; Liu et al. 2016; Di Carlo et al. 2009), the RBC could move only in the flow direction. Different Reynolds numbers ($Re = \frac{\rho D U_{max}}{\mu } \in [0.1,0.5]$) and dimensionless particles positions in the channel ($r^* = \frac{r}{R_c} \in [0.2,0.7]$ with *r* particle radial position and $R_c$ channel radius) were considered to gather a sufficient amount of data. For each considered *Re*, all the seven dimensionless positions were tested, giving a total number of 60 simulations. More specifics about the chosen setup and scale-up strategy can be found in Porcaro and Saeedipour (2024). It should be noted that, since the RBC shear modulus $k_s$ is kept constant throughout the simulation campaign and the internal fluid of the RBC is not simulated, the effect of two parameters that affect RBCs dynamics in flow is not taken into account. The elastic capillary number $Ca_e = \frac{\mu \dot{\gamma }D_{RBC}}{k_s}$ will become proportional to the Reynolds number, since only the shear rate $\dot{\gamma }$ will be changing, while the inner-to-outer viscosity ratio $\lambda$ is considered to be constant through a global volume conservation energy (Kotsalos et al. 2019).

**Fig. 9:**
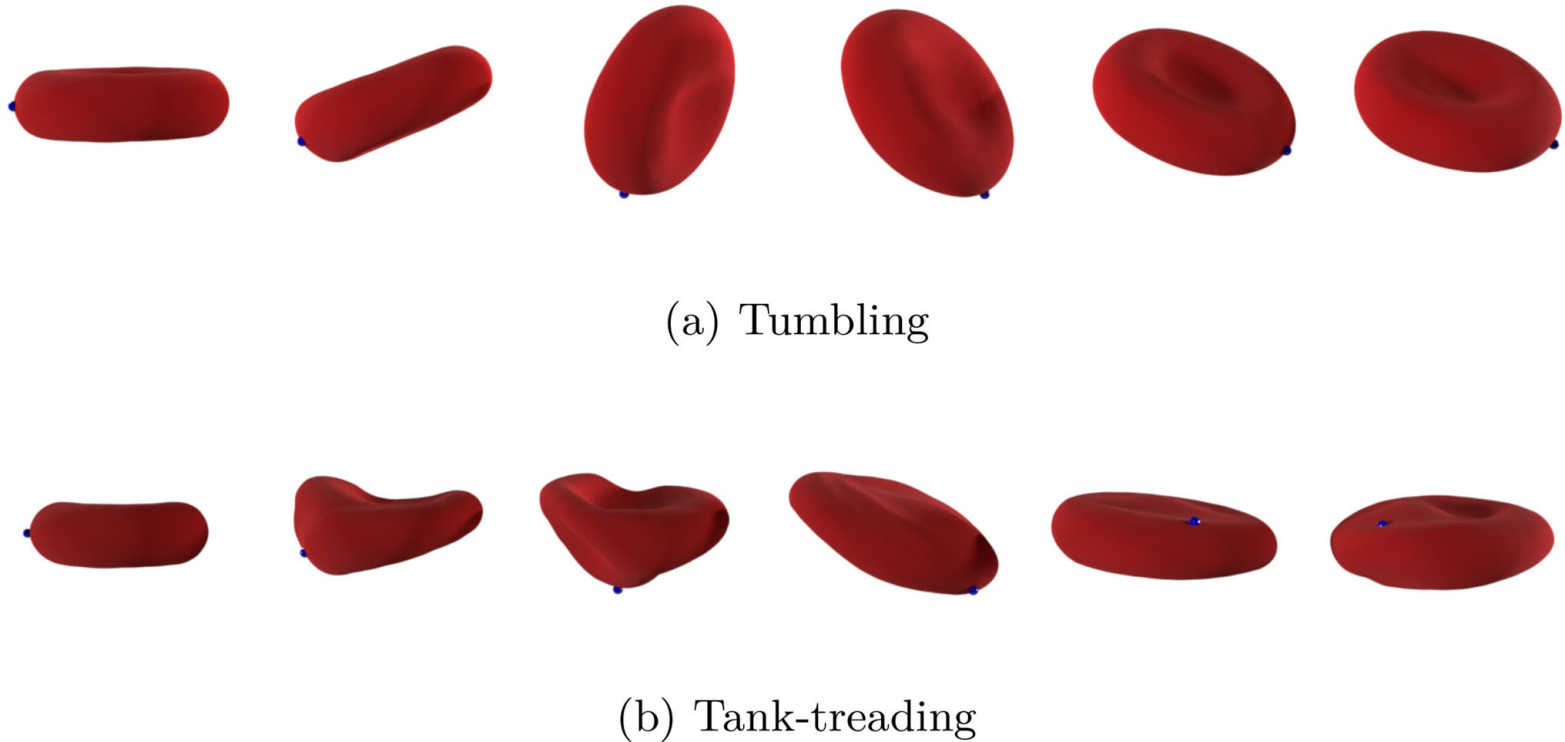
Snapshots of RBC resolved simulation illustrating two different flow modes: **a** tumbling and **b** tank treading. The blue dot is used as a reference and represents a vertex on the npFEM membrane representation of the RBC

**Fig. 10:**
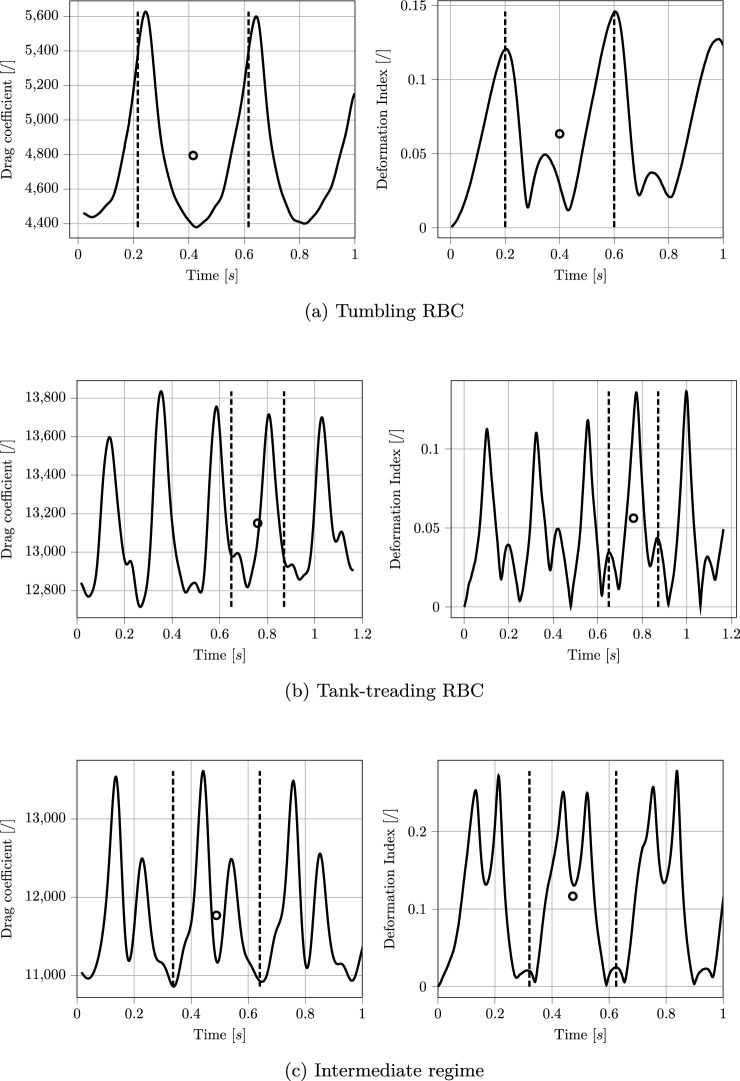
Temporal variation of the drag coefficient $C_d$ and deformation index *DI* for different RBCs flow modes. The vertical dashed lines represent the detected periodicity in the plots, while the circles indicate the corresponding average value for each period. Plots belong to cases with different *Re*

Depending on the flow conditions, two major flowing modes could be identified: (i) tumbling and (ii) tank treading (Fischer et al. 1978; Tran-Son-Tay et al. 1984; Abkarian et al. 2007; Fischer 2007; Mauer et al. 2018; Faghih and Sharp 2019). Figure 9 illustrates these modes, showing the membrane movement over time during a simulation. Tumbling RBCs will fully rotate around their center of mass, while in the case of tank treading, only the external membrane will spin, with the RBC keeping a more or less constant inclination with respect to the flow direction. Intermediate flow modes were also observed, including tumbling and tank treading combinations and more complicated patterns. It has to be highlighted that all of these motions exhibit periodic behavior in terms of RBC shape and orientation. This was particularly relevant for the modeling part of this work since it allowed considering the period average of each measured quantity as a final value for each couple of *Re* and $r^*$. This was necessary since the development of the model needs a direct correspondence between the flow conditions (represented by the pair of values $(Re, r^*)$ and the computed variable. Fig. 10 shows plots of the measured drag coefficient $C_d$ and deformation index *DI* for different flow modes, together with the computed average values. It should be noted that, differently from Sect.  3.2, the 3D version of the deformation index is defined as $DI = \frac{L - B}{L + B}$, with *L* and *B*, respectively, the longest and shortest axis of the RBC. A similar trend was observed for the other measured quantity, the lift coefficient $C_l$. A specific consideration is needed for the RBC orientation instead: only tank treading RBCs would show a preferred orientation during the periodic motion, with their angle relative to the flow direction oscillating around a certain mean value. The average of this angle for tumbling RBCs would be zero, while in the intermediate case it would be difficult to assign one single value over a period due to the unsteadiness of the RBC motion. For this motivation, modeling of RBCs orientation was postponed to future studies, including more than a single RBC in the resolved simulation phase. In that situation, previous literature (Bitbol and Leterrier 1982; Fujii et al. 1999; Lanotte et al. 2016; Rovas et al. 2023) suggests that RBCs will tend to orient in the flow direction, possibly because of steric effects. Therefore, closure models for RBCs orientation in flow are not included in this work.

**Fig. 11:**
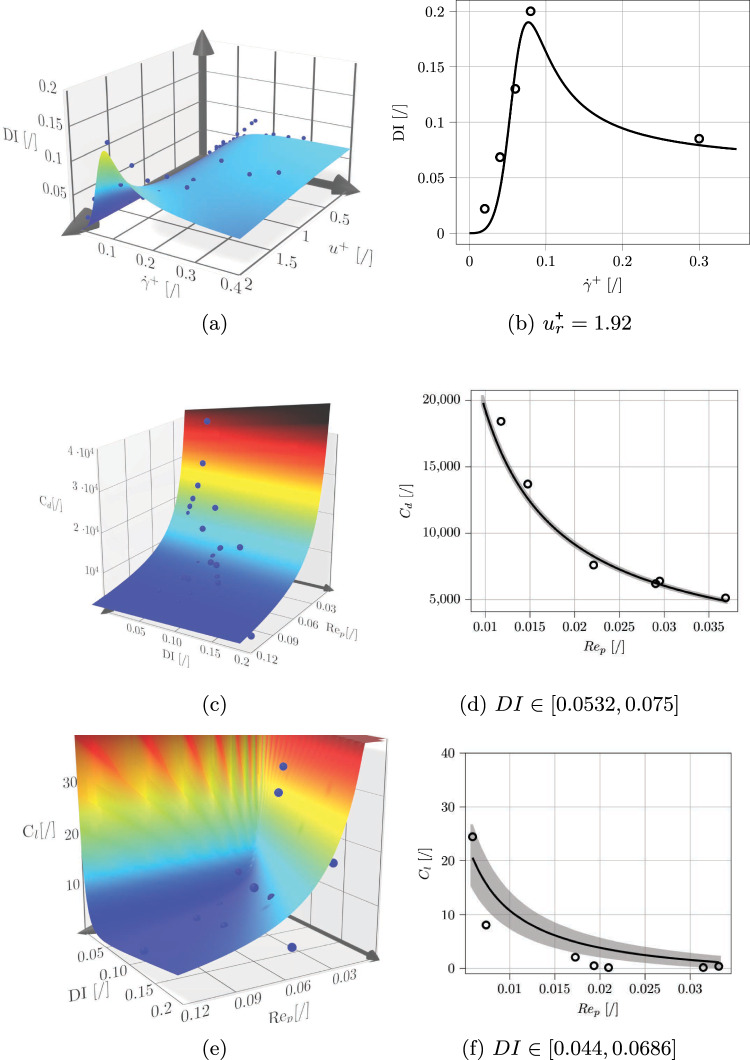
Plots of resolved data and interpolated expressions for the three different considered variables: first row *DI*, second row $C_d$, and third row $C_l$. The left column represents data as blue spheres and models as 3D surfaces. The right column depicts 2D slices for the same correlations, with: black circles as data, black curves as function, and gray shaded areas as function varying with the other independent variable

Once the resolved dataset was acquired, a genetic programming algorithm based on the Python library *gplearn* was employed to find the needed correlations for unresolved simulations. The three quantities to be modeled were the deformation index and the drag and lift coefficients. First, the deformation index was found to be a function of two dimensionless quantities: the dimensionless shear rate $\dot{\gamma }^{\texttt {+}} = \frac{\dot{\gamma }R_c^2}{\nu }$ (with $\dot{\gamma }= \frac{\partial u}{\partial r}$ and $\nu = \frac{\mu }{\rho }$) and the dimensionless velocity $u_{r}^{\texttt {+}} = \frac{u_p - u}{u_{av}}$. The expression reads:

21 $$\begin{aligned} DI = \frac{\dot{\gamma }^{\texttt {+}^3}}{\dot{\gamma }^{\texttt {+}^3}(10 + u_r^{\texttt {+}^3}) + 0.117 \dot{\gamma }^{\texttt {+}^2}(u_r^{\texttt {+}} - u_r^{\texttt {+}^4}) + 0.0026} \nonumber \\ \end{aligned}$$

The drag and lift coefficients were found to be dependent on DI itself, as well as on particle Reynolds number $Re_p = \frac{d_p(u_p-u)}{\nu }$, with $d_p$ particle diameter:

22 $$\begin{aligned} &  C_d(DI, Re_p) = \frac{169 Re_p^2 (DI+1) + 0.00146 }{Re_p ^ 3} \end{aligned}$$

23 $$\begin{aligned} &  C_l(DI, Re_p) = \frac{-3.19 DI^4 }{Re_p} + \frac{2.48 DI }{Re_p} + \frac{0.0008 Re_p }{DI^3} \nonumber \\ \end{aligned}$$

The resolved data and the results of the regression procedure are visualized in Fig. 11. The final equations for the drag and lift forces read:

24 $$\begin{aligned} &  {\textbf {F}}^{\textbf {f}}_{\textbf {p,drag}} = 0.125~C_d~\rho ~\pi ~d_p^2~|{\textbf {u}} - {\textbf {u}}_{\textbf {p}}|~({\textbf {u}} - {\textbf {u}}_{\textbf {p}}) \end{aligned}$$

25 $$\begin{aligned} &  {\textbf {F}}^{\textbf {f}}_{\textbf {p,lift}} = C_l~\rho ~d_p^4~\left( \dot{\gamma }^2+\left( \frac{u_p - u}{d_p}\right) ^2\right) {\textbf {r}} \end{aligned}$$

with ${\textbf {r}}$ normalized vector in the radial direction.

### Model scope of validity and performance

This section aims to clarify the applicability limits of the developed closures, and ensure a clear understanding of the combined modeling activities of the current work and of Porcaro and Saeedipour (2024). As mentioned in the previous paragraph, 60 simulations were included in the dataset, combining six dimensionless positions ($r^* = \frac{r}{R_c} = [0.2, 0.3, 0.4, 0.5, 0.6, 0.7]$) and five channel Reynolds numbers ($Re = \frac{\rho D U_{max}}{\mu } = [0.1, 0.2, 0.3, 0.4, 0.5]$). This leads to a range of dimensionless shear rates of $\dot{\gamma }^{\texttt {+}} \in [0.02-0.35]$ and a range of particle Reynolds numbers $Re_p \in [0.01 - 0.12]$. Each simulation was averaged over a minimum of three periods to ensure that the collected data was truly a steady-state average. Therefore, each simulation only produced a single data point per each analyzed quantity (deformation index, drag, and lift forces). The applicability of the developed model to setups outside of the aforementioned ranges is not guaranteed. Other possible sources of uncertainty are correlated to parameters such as confinement ratio (ratio between particle and channel radius) and to different geometries (different channel cross-section shape, bifurcations). It should be noted that a full held-out validation of the learned correlations remains beyond the scope of the present study and will be the subject of future work. Once the data were collected, the genetic programming algorithm was run until the stopping criteria were reached for the considered objective metric (which was chosen based on the dataset). Table 1 includes the information about the parameters used for the genetic programming algorithm execution.

**Table 1:** Parameters for the genetic programming regression

	*DI*	$C_d$	$C_l$
Metric	Mean square error	Mean absolute error	Mean square error
Stopping criteria	0.0001	0.0001	0.000001
Population size	5000	5000	5000
Generations	20	20	20
Tournament size	20	10	10
Probability of crossover	0.9	0.9	0.9
Probability of subtree mutation	0.01	0.01	0.01
Probability of hoist mutation	0.01	0.01	0.01
Probability of point mutation	0.01	0.01	0.01

It has to be pointed out that the deformation index calculation is the main part of the model, since the other two developed expressions are directly dependent on it. This is coherent with the physics of RBCs, since their dynamics is strongly connected to deformation (Hur et al. 2011; Catarino et al. 2019; Porcaro and Villone 2023). If one considers the deformation index to be constant (e.g., malaria-infected hardened RBCs), this would lead to drag and lift forces purely dependent on the particle Reynolds number, consistent with the existing literature on rigid particles. It should be noted that, since the correlations were developed on a single RBC level, the hematocrit in all the considered simulations is kept at a low value (around 5%). This allows for better judging of the model performance, since cell-cell interactions and the influence of other RBCs on the computed correlations were not optimized yet, and are left for future studies. This will be better discussed in the conclusion. Nevertheless, it has to be highlighted that, in principle, unresolved simulations of RBCs could be easily performed with a physiological hematocrit of 40%. Furthermore, Porcaro and Saeedipour (2024) have proven that correlations based on a single RBC resolved approach can produce reliable results until a hematocrit of 15%. It has to be noted that this threshold only refers to the specific cases presented in this work and in Porcaro and Saeedipour (2024), since it is hard to define a universal number after which cell-cell collisions become dominant. This strengthens the choice of keeping the hematocrit in the remaining part of the work at such a low value, which most likely ensures that cell-cell interactions are not the main part of the RBCs’ physics. Nevertheless, future modeling activities must include such interactions in a way that makes this distinction between a dilute and a dense system automatically taken care of by the model (e.g., corrections to the closure models that are proportional to the local hematocrit, similar to what has been proposed in literature for rigid particles (Beetstra et al. 2007). All simulations were performed on a local cluster at the Johannes Kepler University which employs a quad-core X5570 Xeon CPUs @2.93GHz, with a number of cores varying from 8 to 32 depending on the number of RBCs involved in the cases, and with an execution time that could vary from a few hours to one week for the most computational demanding setups. For a more accurate review of the method’s performance, the reader is referred to Porcaro and Saeedipour (2024). Once the closure expressions have been developed, they could be used in Eq. (18) as part of the fluid-particle interaction force in the unresolved CFD–DEM approach to investigate RBC-laden flows. As the first validation case, the RBC-laden flow in a straight cylindrical channel is simulated, and the results are compared to experimental data and previous numerical simulations. Then, two additional case studies are performed to further explore the potential of the developed upscaling strategy in real-scale bio-microfluidic applications.

### Straight cylindrical channel

Blood is known to experience the Fåhraeus–Lindqvist effect when circulating in micro-channels (Fåhraeus and Lindqvist 1931). RBCs are pushed toward the channel central axis by the deformability-induced lift force and by volume exclusion effect, leaving a layer of pure plasma close to the wall, known as cell-free layer. As a consequence, the local hematocrit measured at the exit of the channel (called discharge hematocrit $Ht_d$) is normally higher than the original channel hematocrit $Ht_c$. This effect is triggered by the higher velocity experienced by the RBCs in the channel center, which in turn increases their measured flux at the channel outlet. Another secondary effect of cross-stream migration is the decrease in the apparent viscosity of blood when compared to pure plasma due to the lubricating effect. This is estimated through the relative apparent viscosity $\mu _{rel} = \frac{\mu _{blood}}{\mu _{plasma}}$. Since a periodic boundary condition was applied, each simulation was run until a steady-state result was reached for $Ht_d$ and $\eta _{rel}$. Both were measured based on the volumetric fluxes of whole blood ($Q_{blood}$) and RBCs ($Q_{RBCs}$) at the outlet face of the channel. $Ht_d$ is given by the ratio between $Q_{RBCs}$ and $Q_{blood}$, while $\eta _{rel}$ is the ratio between the theoretical flux of pure plasma flowing in the same conditions as the simulation and $Q_{blood}$. Pries et al. (1992) gathered the experimental literature on these two quantities and performed some additional experiments to derive empirical formulas for their calculation, based on $Ht_c$ and channel diameter *D*:

26 $$\begin{aligned} &  Ht_d = -\frac{X}{2-2X}+\left[ \left( \frac{X}{2-2X} \right) ^2 + \frac{Ht_c}{1-X} \right] ^{0.5} \end{aligned}$$

27 $$\begin{aligned} &  X = 1 + 1.7e^{-0.35D} - 0.6e^{-0.01D} \end{aligned}$$

and

28 $$\begin{aligned} &  \mu _{rel} = 1 + (\mu _{rel, 0.45} - 1) \frac{(1- Ht_d)^C - 1}{(1- 0.45)^C - 1} \end{aligned}$$

29 $$\begin{aligned} &  \mu _{rel, 0.45} = 3.2 + 220e^{-1.3D} - 2.44e^{-0.06D^{0.645}} \end{aligned}$$

30 $$\begin{aligned} &  C = (0.8 + e^{-0.075D}) \left( -1 + \frac{1}{1+10^{-11}D^{12}}) \right) \nonumber \\ &  \qquad \ + \frac{1}{1+10^{-11}D^{12}} \end{aligned}$$

**Fig. 12:**
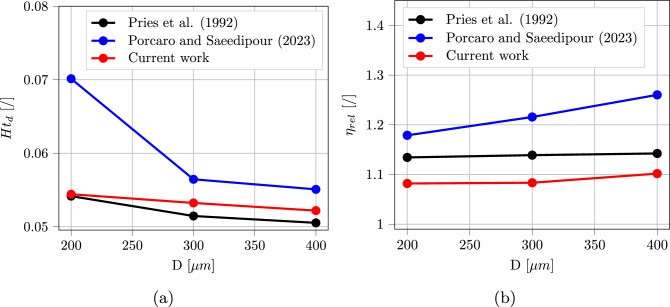
Discharge hematocrit $Ht_d$ and relative apparent viscosity $\mu _{rel}$ as a function of channel diameter *D* for a channel hematocrit $Ht_c = 0.05$: comparison among (Pries et al. 1992) experimental correlation, Porcaro and Saeedipour (2024) and current work

**Fig. 13:**
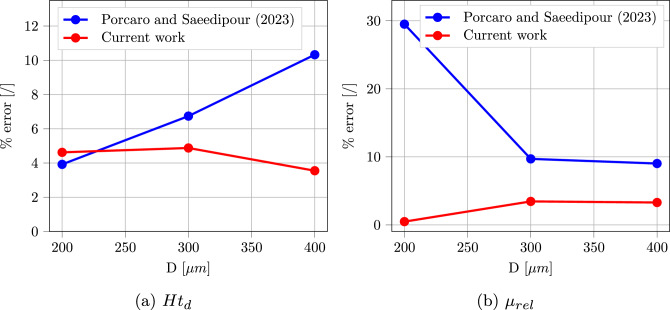
Relative percentage error with respect to the correlation data from Pries et al. (1992) as a comparison between (Porcaro and Saeedipour 2024) and current work: (a) error related to $Ht_d$ and (b) error related to $\mu _{rel}$

These correlations can be used to validate the accuracy of the unresolved CFD–DEM approach in predicting blood macroscopic characteristics. Authors have already demonstrated that a correct description of drag and lift forces acting on the RBCs is crucial in order to correctly reproduce the experimental trends (Porcaro and Saeedipour 2024). In this previous work, two sets of simulations were compared: one using classical force models developed for rigid spheres, and one with RBC-specific models. The latter showed improved agreement with the experimental data, confirming the importance of the elaborated framework. Simulations are repeated in this work with updated closure models to show additional improvement related to the usage of a better resolved simulation dataset, which allows the inclusion of further parameters in the model such as the Deformation Index (Fig. 12 and  13.) For both the considered macroscopic variables, the current approach reduces the percentage error $\%_{error} = 100 \cdot \frac{|x_{sim} - x_{Pries}|}{x_{Pries}}$ to a maximum of 5% in all the simulated cases. It should be also noted that, differently from the previous work, the error remains approximately constant in all the considered ranges, leading to more reliable results.

### Design of bifurcated microfluidic channel

**Fig. 14:**
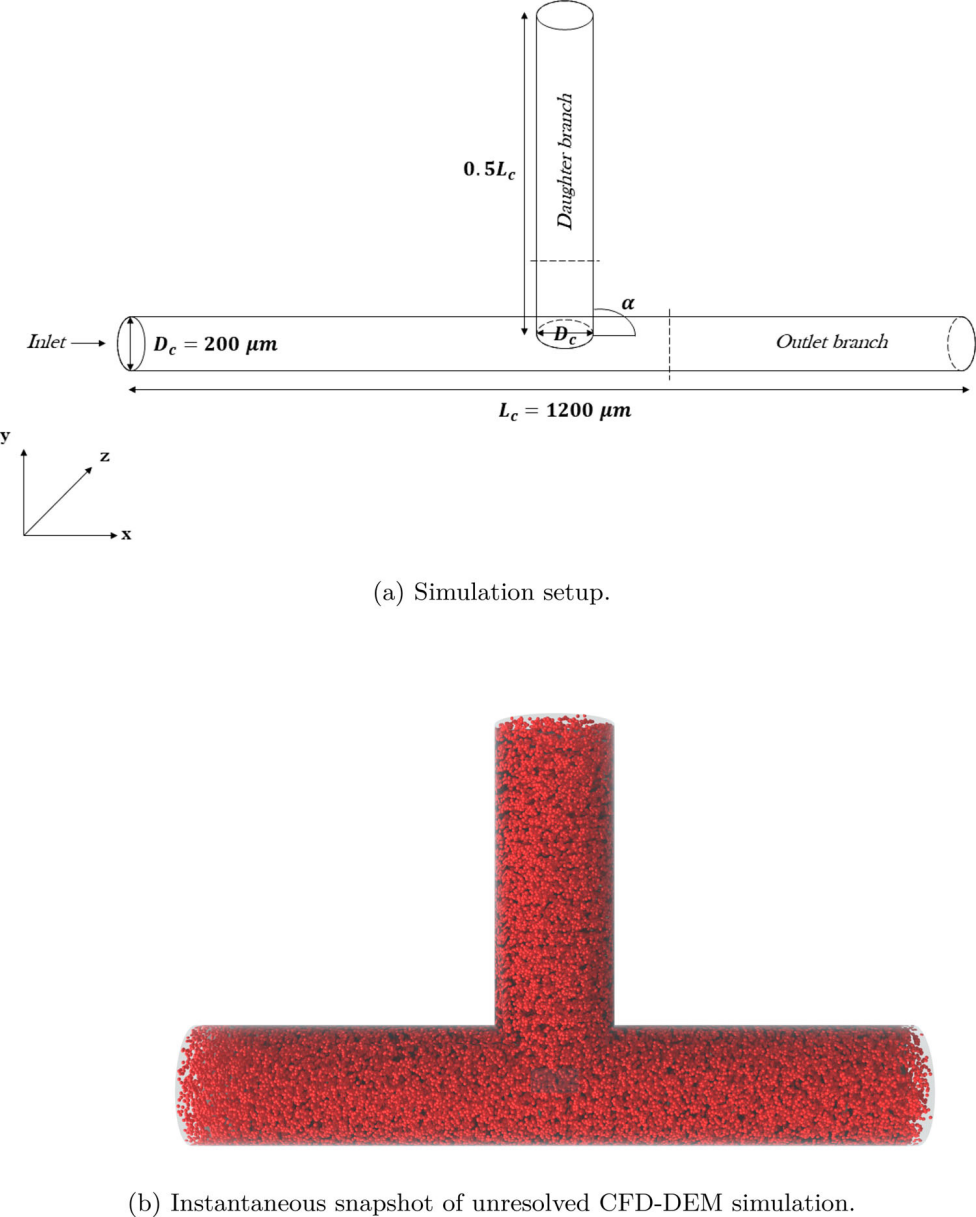
Bifurcated microfluidic channel **a** simulation setup, and **b** an instantaneous snapshot of unresolved CFD–DEM simulation of RBC-laden flow. Dashed lines indicate the $200~\mu m$ distance from the bifurcation in each channel where the hematocrit profile was measured to compute $S_H$

The derivation of Eq. (21) to compute RBCs deformation index in flow has the additional advantage of allowing its usage to perform a statistical analysis on the deformation of the RBC population in microfluidic channels. Excessive deformation can lead to hemolysis, namely the leakage of hemoglobin from the RBC core to plasma due to the formation of micro-pores in the membrane. This needs to be strongly avoided in biomedical tools due to risks going from invalidation of clinical tests to serious health hazards for the patient (Peng et al. 2020; Guo et al. 2022). To showcase the potential utility of the unresolved CFD–DEM approach in this context, simulations of a bifurcated microfluidic channel with different angles of bifurcation $\alpha$ were performed. The setup is inspired by the experimental work of Stathoulopoulos et al. (2024) (although with some differences), and it is illustrated in Fig. 14.

**Fig. 15:**
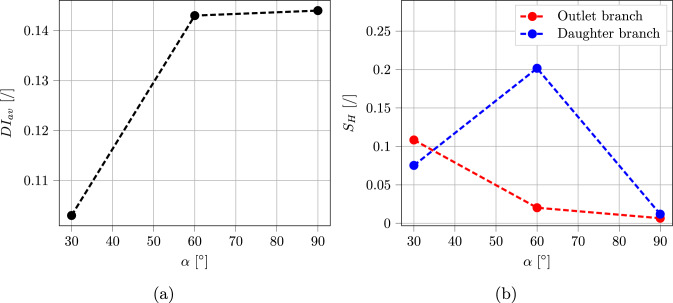
Variation of blood flow quantities with bifurcation angle $\alpha$: **a** averaged deformation index $DI_{av}$, and **b** hematocrit skewness $S_H$

**Fig. 16:**
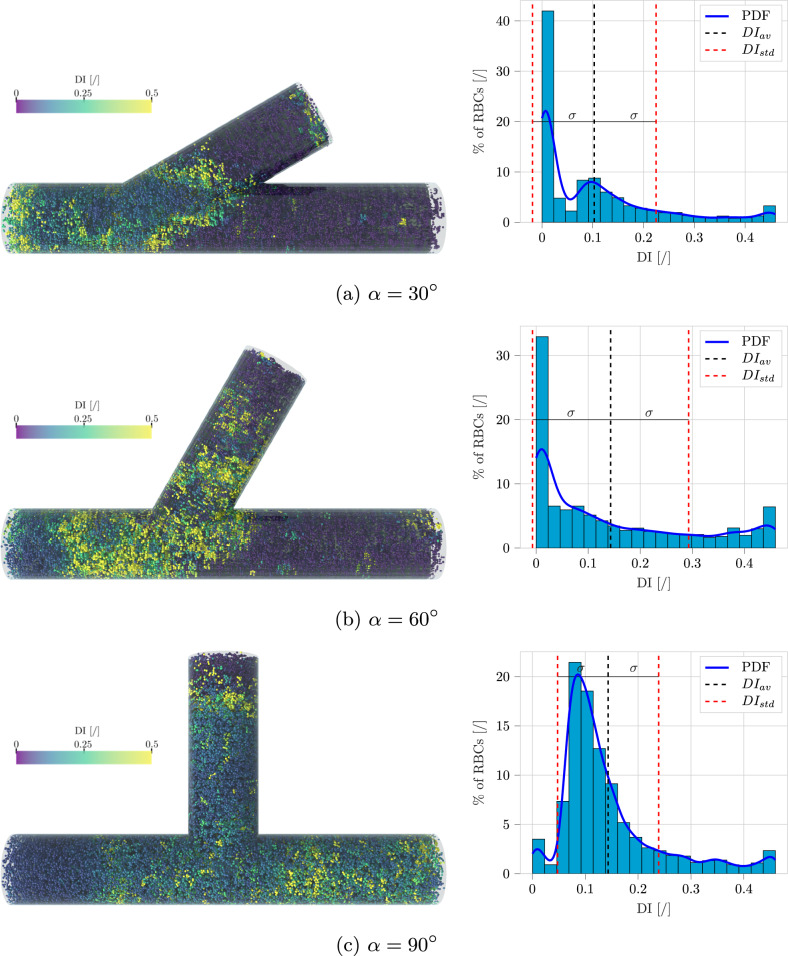
Comparison among channels with different bifurcation angles $\alpha$. Left column: RBCs colored based on their deformation index. Right column: Histograms of *DI* of the RBCs population

The bifurcation angle $\alpha$ was varied among 30, 60, and 90 degrees to understand which setup had the major impact on RBCs deformation. Figure 15 shows simulation results for the different considered angles. The setup with $\alpha = 30^\circ$ exhibits a lower average deformation index compared to the other two, therefore should be preferred when trying to reduce hemolysis occurrence. A further analyzed quantity was the hematocrit skewness index $S_H$, which defines the asymmetry of the hematocrit profile along a specified cut (see Fig. 14):

31 $$\begin{aligned} S_H = \left| \frac{\int _0^{0.5} H_t (w)~dw}{\int _{-0.5}^{0.5} H_t (w)~dw} -0.5 \right| \end{aligned}$$

with *w* normalized coordinate along the cut direction (Stathoulopoulos et al. 2024). In line with previous experimental literature (Sherwood et al. 2014; Stathoulopoulos et al. 2024), $S_H$ was found to be higher in the daughter branch compared to the outlet branch for values of $\alpha = 60^\circ$ and $\alpha = 90^\circ$. The trend is inverted in the case of $\alpha = 30^\circ$, which was not covered in previous works. Figure 16 illustrates snapshots of the three different performed simulations, together with distributions of the deformation index *DI* for the RBC population. This allows to individuate zones where the cells are subjected to higher deformation, and to gather another important information: even if the two devices with $\alpha = 60^\circ$ and $\alpha = 90^\circ$ share the same average *DI*, the one with $\alpha = 60^\circ$ has a higher number of mildly deformed RBCs.

### Stenosed channel

**Fig. 17:**
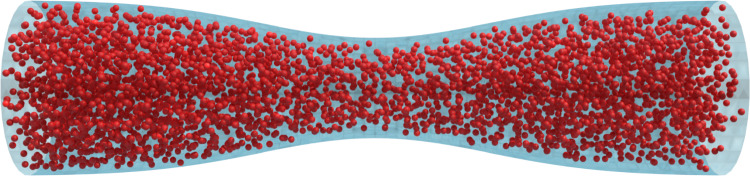
Unresolved CFD–DEM simulation of a stenosed channel with a diameter of $150~\mu m$ and a minimum length of $85~\mu m$. A constant RBC flux is injected as an inlet to keep the hematocrit around 5%

**Fig. 18:**
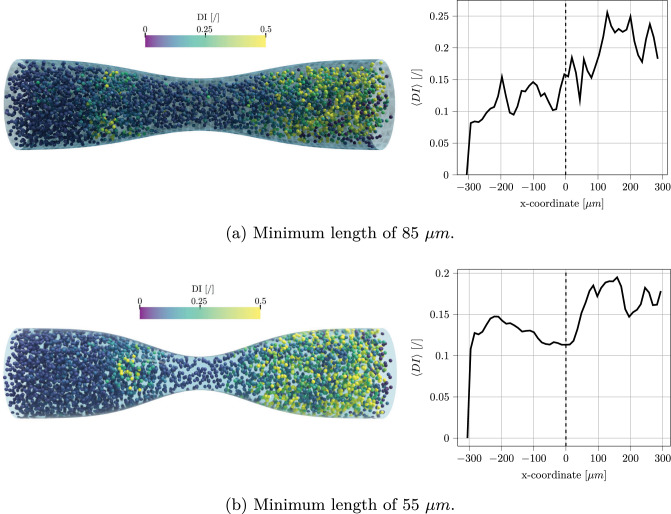
Comparison between two channels with a minimum length of $85~\mu m$ and $55~\mu m$. Left column: RBCs colored based on their deformation index. Right column: spatially averaged $\langle DI \rangle$ along with the channel flow direction

The stenosed channel geometry depicted in Sect.  3.2 is increased ten times in size to study the effect of the degree of stenosis on a population of several RBCs (see Fig. 17). This kind of simulation could be useful for the study of vein occlusion caused by thrombosis. The channel diameter was $150~\mu m$, and the two minimum channel sizes considered were $85~\mu m$ and $55~\mu m$. Figure 18 shows that in both cases there is a strong increment of the RBC deformation after the contraction. This information can help understand how the dimension of the thrombus (which increases the degree of stenosis of the channel) can lead to additional stress on the cell membrane, enlarging the amount of deformation and thus the cell damage. As mentioned at the start of this section, the hematocrit was kept intentionally low, therefore no actual occlusion was observed in this case. Future studies will focus on cell-cell interactions to overcome current limitations and allow simulations of stenosed channels with physiological hematocrit, leading to further advancement in the topic.

## Conclusions

This work presents an upscaling strategy for the simulation of RBCs in flows with low Reynolds number. A dataset based on resolved LBM-npFEM simulations is obtained over a range of different flow conditions. Afterward, this dataset is used to derive closure models for unresolved CFD–DEM simulations, including expressions for RBCs deformation index and drag and lift force. Subsequently, the unresolved method is used in different applications, such as validation of experimental data for blood macroscopic characteristics, design of a microfluidic bifurcated device, and study of a stenotic channel. To the best of the authors’ knowledge, the presented methodology is one of the first attempts to bridge the scale separation gap in cell-level blood simulations that led to the development of case-independent correlations for upscaling, and it allows large-scale cell-level simulations potentially involving up to millions of RBCs.

Limitations of the current approach are strongly related to the dimensions of the dataset built from resolved simulations. The developed expressions have shown the ability to correctly predict RBCs behavior in flow beyond the range of the dataset parameters; however, as the deviation from this range increases, the probability of reduced accuracy also grows. On a single RBC level, a wider parameter space needs to be explored to correctly model the RBC behavior in flow under different conditions (such as higher Reynolds or different RBC deformability). Besides, parameters such as the capillary number and the inner-to-outer viscosity ratio can significantly alter RBCs dynamics and should be included in the modeling phase, especially in applications in which their influence is proven to be non-negligible, e.g., Malaria infection diagnosis (Ademiloye et al. 2017). It should also be noticed that, until a full description of the RBCs underlying physics is achieved in the model, changes in the geometry (such as different confinement ratios, cross-section shape and bifurcations) may reduce the accuracy of the proposed approach.

Furthermore, it is necessary to account for the presence of other RBCs already in the modeling phase for two different reasons. First, modification of the derived closure laws based on the presence of other RBCs in the surroundings of the considered one is required. This will alter the flow patterns and thus change the computed correlations. A similar problem has already been faced in the context of CFD–DEM simulation of rigid spheres (Beetstra et al. 2007). Furthermore, cell-cell interactions require further understanding in order to optimize collisions and cohesion models for unresolved RBCs. Besides, a recent work by Valtchanov et al. (2025) has proven the importance of collisions for phenomena such as hemolysis. All of the aforementioned topics will be the object of future studies, together with the inclusion of other components of blood (such as platelets and white blood cells) in the CFD–DEM simulation tool. Additionally, the accuracy of different methods for the fitting procedure on the obtained data has to be tested. This was not the focus of the current paper, but it will become a relevant topic when extending the parameter space to higher dimensions.

Nevertheless, this upscaling approach entails great potential in different applications in the biomedical field. Besides aiding the design of microfluidic devices and organs on a chip, it can help clarify mechanisms behind platelet adhesion and thrombus formation on a large scale, once correctly tuned through small-scale resolved simulations (Ye et al. 2020). Similarly, the possibility of coarse-graining different physical variables, such as the ones involved in species transport, discloses the opportunity of applying the same strategy to problems such as hemolysis prediction and usage of RBCs as biocompatible drug carriers.

## Data Availability

No datasets were generated or analyzed during the current study.
